# The Physician's Diary for 1876

**Published:** 1876-01

**Authors:** 


*The Physicians Diary for* 1876.—Containing a visiting list diary and daily memoranda, obstetric and vaccination records, etc., etc., with tables of incompatibles, thermometry in diseases, Marshall Hall's ready method, phases of the moon, poisons and their antidotes, list of medicines by Tilden & Co., with doses and formulæ for reducing their fluid extracts to tinctures, syrups, &c. Published at the office of the "*Journal of Materia Medica,*" New Lebanon, N. Y., 1876, in a very neat and attractive form, with gilt edges, and on excellent paper.

